# The Effectiveness of Digital Health Interventions in the Management of Musculoskeletal Conditions: Systematic Literature Review

**DOI:** 10.2196/15617

**Published:** 2020-06-05

**Authors:** Stephanie Hewitt, Ruth Sephton, Gillian Yeowell

**Affiliations:** 1 St Helens Therapy Department North West Boroughs Healthcare NHS Foundation Trust Merseyside United Kingdom; 2 Department of Health Professions Manchester Metropolitan University Manchester United Kingdom

**Keywords:** musculoskeletal pain, physical functional performance, health communication, online intervention, web-based intervention, mobile phone

## Abstract

**Background:**

Musculoskeletal conditions are the second greatest contributor to disability worldwide and have significant individual, societal, and economic implications. Due to the growing burden of musculoskeletal disability, an integrated and strategic response is urgently required. Digital health interventions provide high-reach, low-cost, readily accessible, and scalable interventions for large patient populations that address time and resource constraints.

**Objective:**

This review aimed to investigate if digital health interventions are effective in reducing pain and functional disability in patients with musculoskeletal conditions.

**Methods:**

A systematic review was undertaken to address the research objective. The review was conducted in accordance with the Preferred Reporting Items for Systematic Reviews and Meta-Analyses guidelines. The review protocol was registered with the International Prospective Register of Systematic Reviews before commencement of the study. The following databases were searched: Medical Literature Analysis and Retrieval System Online (MEDLINE), Excerpta Medica database (EMBASE), Cumulative Index to Nursing and Allied Health Literature, and Scopus from January 1, 2000, to November 15, 2019, using search terms and database specific−medical subject headings terms in various combinations appropriate to the research objective.

**Results:**

A total of 19 English language studies were eligible for inclusion. Of the 19 studies that assessed musculoskeletal pain, 9 reported statistically significant reductions following digital intervention. In all, 16 studies investigated functional disability; 10 studies showed a statistically significant improvement. Significant improvements were also found in a range of additional outcomes. Due to the heterogeneity of the results, a meta-analysis was not feasible.

**Conclusions:**

This review has demonstrated that digital health interventions have some clinical benefits in the management of musculoskeletal conditions for pain and functional disability. Digital health interventions have the potential to contribute positively toward reducing the multifaceted burden of musculoskeletal conditions to the individual, economy, and society.

**Trial Registration:**

PROSPERO CRD42018093343; https://www.crd.york.ac.uk/prospero/display_record.php?RecordID=93343

## Introduction

### Background

Musculoskeletal conditions are the second greatest contributor to disability worldwide and have substantial individual, societal, and economic implications [1,2]. The term musculoskeletal conditions is a broad term used to describe a large number of conditions that affect bones, joints, and soft tissues [3]. Musculoskeletal conditions comprise over 100 different disorders, diseases, and syndromes, most of which affect people’s ability to carry out normal activities and impact their quality of life. The most prevalent of these conditions are low back pain (LBP) and osteoarthritis (OA) [3]. Musculoskeletal conditions account for 30% of general practitioner consultations in England and are associated with related comorbidities, including diabetes, depression, and obesity [4]. For the individual, the most common symptoms include pain, aching, stiffness, fatigue, reduced physical functioning, and loss of dexterity [3]. Treating musculoskeletal conditions is estimated to cost the United States US $213 billion [5] and costs the UK economy £10.2 billion (US $12.62 billion) in direct costs to the National Health Service [3].

Due to the prevalence and growing burden of musculoskeletal disability, an integrated, strategic approach that provides effective and accessible models of health service delivery on a population level is urgently required [2,6]. The use of mobile and wireless digital health interventions is one possible solution to deliver this objective [7]. Digital health interventions provide opportunities to tackle health system challenges and offer the potential to enhance the quality and sustainability of musculoskeletal health services [8]. The World Health Organization has recently published guidelines that classify digital health interventions in an attempt to standardize the vocabulary used within the diverse communities working in digital health [8]. In cognizance of this, digital health interventions in this study applies to interventions for clients, with all digital health interventions being delivered as apps, via websites or via web-based software [8].

Digital health interventions can provide high-reach, low-cost, readily accessible, and scalable patient education and self-management interventions that address time and resource constraints for musculoskeletal populations, delivered via apps or web-based platforms [6]. However, there are problems facing the implementation of digital health interventions [9]. A common problem is the failure of agreement on what constitutes appropriate evaluation before widespread rollout [9,10]. In addition, there is tension between the dynamic development of digital interventions and the slow transition into clinical practice from more conventional clinical trial outcomes [9-11]. If digital interventions are to be utilized as therapeutic interventions for musculoskeletal conditions, clinicians and service users need to have confidence in their effectiveness [10,11]. Ultimately, the development and utilization of digital health interventions in a therapeutic capacity for musculoskeletal conditions need to work toward reducing the burden of musculoskeletal-related disability.

### Objective

The primary aim of this systematic review was to assess if digital health interventions are clinically effective in reducing pain and functional disability in patients with musculoskeletal conditions. The secondary aim was to explore the content, characteristics, and delivery of digital health interventions in the studies identified to ascertain if there were specific aspects of the interventions, or the population they were targeting, that were associated with beneficial outcomes. To the best of our knowledge, no systematic review has examined the effectiveness of digital health interventions in the management of musculoskeletal conditions.

## Methods

### Overview

A systematic review was undertaken to address the aims of the study. The authors (SH and RS) were assisted in the literature search by an experienced librarian, proficient in searching medical databases. The review was conducted in accordance with the Preferred Reporting Items for Systematic Reviews and Meta-Analyses (PRISMA) guidelines [12]. The review protocol was registered with the International Prospective Register of Systematic Reviews (PROSPERO reference: CRD42018093343) before commencement of the study. However, deviations from the protocol were required following the pilot study. This was related to the inclusion of the PsycINFO database, which yielded no useful results and, as such, was not included in the main search.

### Search Strategy

A systematic search of the following databases was conducted: MEDLINE, EMBASE, Cumulative Index to Nursing and Allied Health Literature, and Scopus from January 1, 2000 to November 15, 2019. The search was conducted on November 15, 2019. Abstract and subject-heading search terms pertinent to the study aims were developed and finalized jointly by the 3 authors (SH, RS, and GY) following background literature searches and a pilot study. Search terms and database specific−medical subject heading terms were used in various combinations (Multimedia Appendix 1). Boolean operators “OR” and “AND” were used to combine search terms.

**Figure 1:**
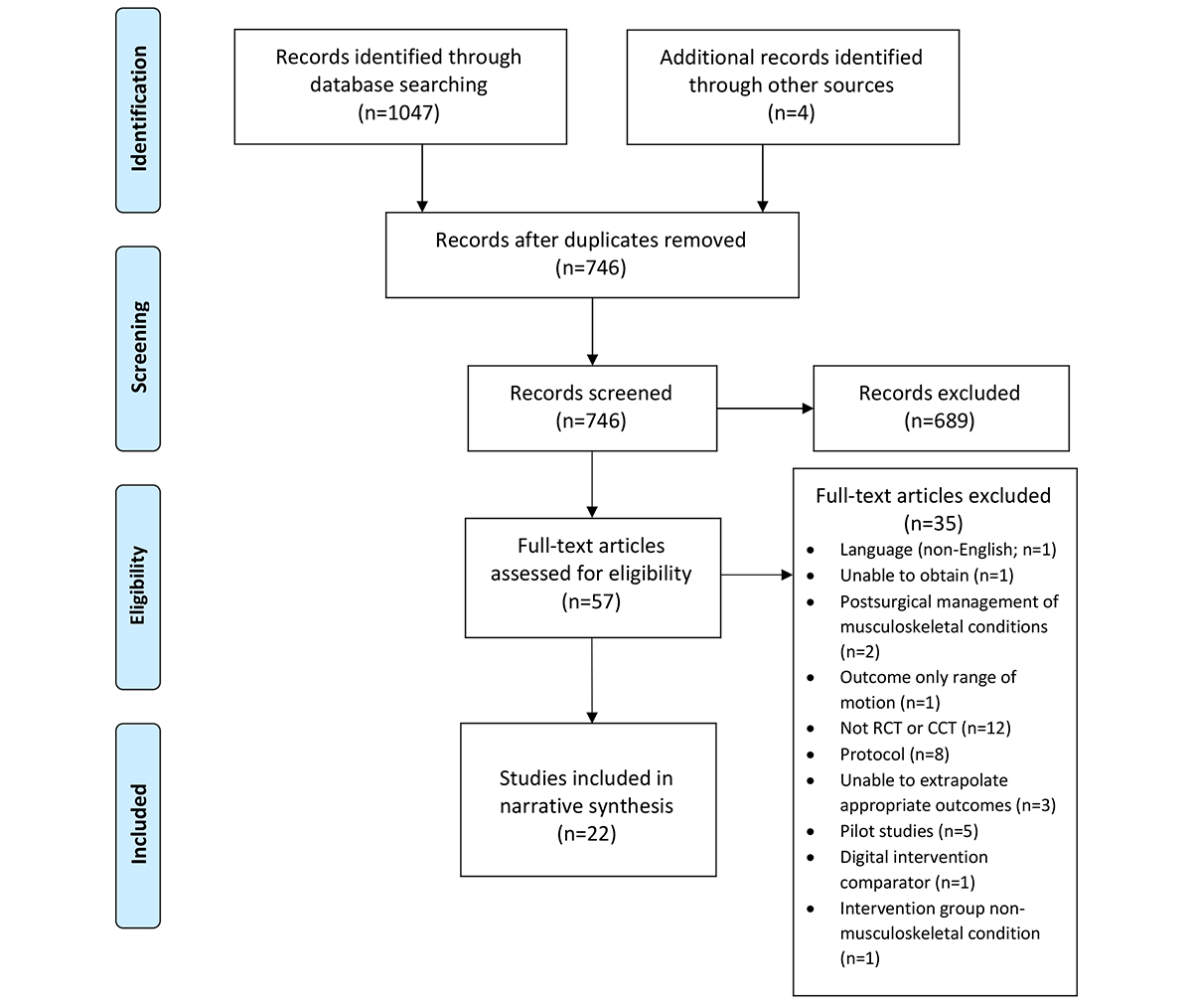
Preferred Reporting Items for Systematic Reviews and Meta-Analyses flow diagram indicating search strategy. CCT: controlled clinical trial; RCT: randomized controlled trial.

Eligibility criteria were guided by the population, intervention, comparator, outcome, and study design framework [13] (Textbox 1). Titles and abstracts were reviewed for eligibility by 2 reviewers (SH and RS). Full-text papers were obtained and independently screened against the eligibility criteria by the same reviewers (SH and RS), and any disagreements were resolved through discussion. A third reviewer (GY) was available to resolve disagreements; however, this was not required. Manual searching of the reference lists was undertaken to identify any additional studies. The PRISMA flowchart details the search strategy for this review (Figure 1).

**Eligibility criteria.**

- Inclusion criteria
  - Population
    - Adults (older than 18 years) with musculoskeletal conditions (acute, subacute, and chronic)
    - Setting: Anywhere patient has access to the internet
  - Intervention
    - Any form of digital-based intervention/treatment delivered by any digital means (eg, website or app) over any time frame.
    - Digital health intervention: For the purpose of this review, *digital health interventions* refers to *interventions for clients*, including targeted client communication; personal health tracking; and on-demand information services delivered by apps, web-based software, or websites. It includes any intervention accessed through a computer (work or home), or smartphone, or other hand-held device, and it includes desktop computer programs or apps that provide self-management information and can be used online or offline. The intervention must function without the need for directive input from a health professional. They must also be *interactive*, which we define as requiring contributions from program users (eg, entering personal data and making choices), which alter pathways within programs to produce tailored material and feedback that is personally relevant to users.
  - Comparator
    - The stated intervention(s) compared with waiting list control (no intervention) or alternative (*standard*) means of delivery (eg, face-to-face, class-based, and printed materials/hand-outs), nondigital self-management interventions (eg, leaflets), and noninteractive digital (eg, web page of flat copy).
  - Outcomes
    - Any positive or adverse health-based outcome and/or predictive indicators assessing pain and/or physical functioning/disability.
  - Secondary outcomes
    - Any positive or adverse health-based outcome and/or predictive indicators assessing patient knowledge and understanding, self-efficacy, catastrophizing, and empowerment. In addition, assess for any correlation between specific aspects of digital interventions and specific outcomes.
  - Study design
    - Randomized controlled trials in English.
- Exclusion criteria
  - Population
    - Nonmusculoskeletal pathology; postsurgical management, for example, following anterior cruciate ligament repair; and post knee replacement
    - Papers pertaining to (chronic) pain, where it is not possible to extrapolate information specifically relating to (chronic) pain of musculoskeletal origin
    - Papers that examined musculoskeletal pain as a *result* of computer use
  - Intervention
    - Papers where digital interventions are used in combination with other methods of intervention of nondigital origin and it is not possible to extrapolate the information pertaining specifically to digital interventions
  - Comparator
    - Any form of digital-based intervention/treatment
  - Outcome
    - Do not assess pain and/or physical functioning/disability
  - Study design
    - Study protocols, case studies/discussion papers, nonrandomized control trials, pilot studies, conference abstracts, and non-English language

### Data Extraction

Data relating to the research aims were independently extracted by the authors (SH,GY, RS). The data extracted included the study setting details (authors, year, and country of origin), the study population (number of participants, age, and gender), intervention details, duration/follow-up period, and outcome measures used (Table 1 and Multimedia Appendix 2). Any misunderstandings and disagreements were addressed through consultation.

All outcome measures and predictive tools (Multimedia Appendix 6) were acknowledged. There were numerous clinical outcome measures, with considerable disparity across the time frames over which interventions were assessed. In addition, a wide range of digital health interventions were used in the studies. According to Cochrane, this diversity across interventions and comparators is not compatible with statistical assessment via meta-analysis; instead, it is more suitable for narrative interpretation [13]. Attempting a meta-analysis with clinically diverse studies risks obscuring genuine differences in effects, resulting in inappropriate conclusions [13]. Furthermore, undertaking a meta-analysis of studies that are at risk of bias may be misleading, as this will compound the errors and produce results that may be interpreted inappropriately as having more credibility [13]. Therefore, for this review, a meta-analysis was not undertaken.

The results are presented in Multimedia Appendix 4. In line with the review’s primary aim, full details of study results are included in the table for all pain and functional disability outcomes. For additional outcomes, only significant between-group differences that were measured at the final time point in the study, are presented.

**Table 1:** Study and participant characteristics.

Reference	Subjects total, N	Subjects, n			Age (years), mean (SD)					Male: female (%)				
		CG^a^	EG^b^		CG		EG			CG		EG		
			EG1	EG2				EG1	EG2				EG1	EG2
Allen et al [14]	350	68	140^c^	142^d^		64.3 (12.2)		65.7 (10.3)^c^	65.3(11.5)^d^		22:78		29:71^c^	31:69^d^
Bennell et al [6]	148	74	74	—^e^		61.5 (7.6)		60.8 (6.5)	—		46:54		42:58	—
Bennell et al [15]	144	71	73	—		61.3 (7.1)		61.2 (7.2)	—		48:52		38:62	—
Bossen et al [16]	199	99	100	—		63 (5.4)		61 (5.9)	—		30:70		40:60	—
Buhrman et al [17]	54	28	26	—		42.9 (10.1)		43.5 (9.8)	—		36:64		27:73	—
Calner et al [18] and Nordin et al [19]	99	44	55	—		42 (11)		44 (10)	—		16:84		14:86	—
Carpenter et al [20]	141	71	70	—		42.5 (10.3)^f^		—	—		17:83^f^		—	—
Chhabra et al [21]	93	48	45	—		41.0 (14.2)		41.4 (14.2)	—		—^e^		—	—
Chiauzzi et al [22]	199	104	95	—		45.05 (11.72)		47.34 (12.23)	—		32:68		33:67	—
Del Pozo-Cruz et al [23-25]	100	50	50	—		45.5 (7.02)		46.83 (9.13)	—		11: 89		15:85	—
Irvine et al [26]	597	199	199^g^	199^h^		—		—	—		37:63		42:58^g^	41:59^h^
Krein et al [27]	229	118	111	—		51.9 (12.8)		51.2 (12.5)	—		86:14		89:11	—
Marangoni [28]	68	23	22^i^	23^j^		—		—	—		—			—
Mecklenberg et al [29]	125	54	101	—		47 (12)		46 (12)	—		74:26		57:43	—
Peters et al [30]		50	112^k^	114^l^		50.6 (10.1)		48.7 (11.5)^i^	47.5 (13.2)^j^		12:88		15:85^i^	17:83^j^
Petrozzi et al [31]	276	54	54	—		50.6 (14.4)		50.1 (12.8)	—		41:59		46:54	—
Shebib et al [32]	177	64	113	—		43 (12)		43 (11)	—		52:48		63:37	—
Toelle et al [33]	94	46	48	—		43 (11.0)		41 (10.6)	—		33:67		27:73	—
Van den Heuvel et al [34]	268	90	97	81		—		—	—		—		—	—

^a^CG: control group.

^b^EG: experimental group.

^c^PT: physical therapy.

^d^IBET: internet-based exercise training.

^e^not recorded.

^f^Total sample. Not recorded for control group and evaluation group.

^g^Fit back.

^h^Alternative care.

^i^CASP: computer-assisted stretching program.

^j^FLIP: facsimile lesson with instructional pictures (hard copy).

^k^iCBT: internet-delivered cognitive behavioral therapy.

^l^PPI: positive psychology intervention.

### Quality Assessment

Methodological quality of the included studies was assessed by the lead researcher (SH) using the Cochrane risk of bias tool (modified) for quality assessment of randomized controlled trials (RCTs) [35]. This tool examines different subsets of bias, including performance, selection, detection, and attrition [36]. In total, 40% of the papers were independently assessed by a second reviewer (RS), and any disagreements were resolved through discussion. A third author was available (GY) should disagreements not be resolved but was not required.

## Results

### Search Results

A total of 1047 papers of potential interest were identified (Figure 1 and Multimedia Appendix 3). Of these, 301 were excluded as duplicates, leaving 746 for title and abstract screening. Following screening, the full texts of 57 papers were obtained and screened for eligibility, resulting in 22 papers eligible for inclusion.

### Description of the Included Studies

Of the 22 papers identified (Figure 1), 3 papers were published in 1 study [23-25], whereas another study published 2 papers [18,19]. Although the study population and intervention were the same in Del Pozo et al’s [23-25], Calner et al’s, and Nordin et al’s [18,19] papers, different outcomes were reported in each publication. Therefore, for the quality assessment, each of these papers was assessed individually using the designated assessment tool. However, for data extraction, the 3 Del Pozo et al’s papers [23-25], and the Calner et al and Nordin et al’s [18,19] papers, have been combined to avoid duplication of the participant outcomes, thus leaving 19 individual studies included in the data extraction and results tables (Table 1, and Multimedia Appendices 2, and 4). Of the 19 included studies, most were from the United States (n=8) [14,20,22,26-29,32]; 3 were from Australia [6,15,31]; 3 were from the Netherlands [16,30,34]; 2 were from Sweden [17-19]; and 1 each from Spain [23-25], Germany [33], and India [21]. One study was published in 2003 [34]; otherwise, all other studies were published between 2010 and 2019.

Of the studies that reported on the gender of participants, all studies except 3 [27,29,32] had a greater number of female participants. All studies included participants with an average age of 35 to 69 years. A wide variety of musculoskeletal conditions were examined by the included studies. In total, 10 studies investigated digital health interventions for LBP [17,20-27,31-33], 3 studies examined musculoskeletal pain [18,19,28,30], 3 studies investigated knee pain [6,14,29], 2 studies examined hip pain only or both knee and hip pain [15,16], and 1 investigated neck/upper limb pain [34].

### Quality Assessment and Risk of Bias

Risk of bias was assessed using the modified Cochrane collaboration tool for assessing risk of bias [35]. Bias is assessed as a judgment (high, low, or unclear) for individual elements (7 domains) from 5 categories: selection (allocation concealment and randomization procedure), blinding (participants/personal and outcome assessors), completeness of data, selective outcome reporting, and other potential sources of bias [35]. Overall, the methodological quality of the included studies was variable (Table 2; refer to the judging criteria described in Multimedia Appendix 5). Of the 22 included studies, 1 achieved a low risk of bias across all 7 domains [15]. A further 6 were unable to blind the participants [6,14,21,23-25,27,31] but achieved a low risk of bias over the remaining 6 domains. Of the remaining 13 studies, a low risk of bias was achieved in 5 or fewer domains. The most consistent domain that failed to achieve a low risk of bias was the blinding of the study participants. The only study that was able to achieve blinding of participants [15] is one where both the control and the experimental group were told they would receive web-based resources and physiotherapy. However, 1 of the groups received only 8 information sheets on the web (flat copies; see Textbox 1) as opposed to the experimental group, which received an interactive digital intervention—pain coping skills training.

**Table 2:** Modified Cochrane collaboration tool for assessing risk of bias (For all domains, if reported Yes, this would indicate a low risk of bias, No would indicate a high risk of bias, and Unclear would indicate an unclear risk of bias).

Reference	Sequence generation	Allocation concealment	Blinding of participants and personnel	Blinding of outcome assessors	Incomplete outcome data	Selective outcome reporting	Other sources of bias
Allen et al [14]	Yes	Yes	No	Yes	Yes	Yes	Yes
Bennell et al [6]	Yes	Yes	No	Yes	Yes	Yes	Yes
Bennell et al [15]	Yes	Yes	Yes	Yes	Yes	Yes	Yes
Bossen et al [16]	Unclear	Yes	No	No	Yes	No	No
Buhrman et al [17]	Yes	Yes	No	Unclear	Yes	Yes	Yes
Calner et al [18]	Yes	Yes	No	Unclear	Yes	Yes	No
Carpenter et al [20]	Yes	Unclear	Unclear	Unclear	Yes	Yes	No
Chhabra et al [21]	Yes	Yes	No	Yes	Yes	Yes	Yes
Chiauzzi et al [22]	Yes	Yes	No	No	Yes	Yes	No
Del Pozo-Cruz et al [23]	Yes	Yes	No	Yes	Yes	Yes	Yes
Del Pozo-Cruz et al [24]	Yes	Yes	No	Yes	Yes	Yes	Yes
Del Pozo-Cruz et al [25]	Yes	Yes	No	Yes	Yes	Yes	Yes
Irvine et al [26]	Unclear	Unclear	No	No	Yes	Yes	No
Krein et al [27]	Yes	Yes	No	Yes	Yes	Yes	Yes
Marangoni [28]	No	No	Unclear	No	Unclear	Yes	No
Mecklenberg et al [29]	Yes	Yes	No	No	Yes	Yes	Yes
Nordin et al [19]	Yes	Yes	No	Unclear	Yes	Yes	No
Peters et al [30]	Unclear	Unclear	Unclear	Unclear	Yes	Yes	Unclear
Petrozzi et al [31]	Yes	Yes	No	Yes	Yes	Yes	Yes
Shebib et al [32]	Yes	Yes	No	No	Yes	Yes	No
Toelle et al [33]	No	No	Yes	No	Yes	Yes	Yes
Van de Heuvel et al [34]	Yes	Unclear	No	Unclear	Unclear	Yes	No

### Pain

All 19 included studies used outcomes that assessed pain. Of these, 9 reported statistically significant improvements in pain [6,15,16,23-26,28,29,32,33], 4 studies [6,15,16,26] assessed pain at more than one time point, and in 2 studies [15,16], improvement was not maintained at the last time point measured. In all, 4 studies [15,16,23-26] reported effect sizes, measured by odds ratio, eta squared, or Cohen d (Multimedia Appendix 4). The findings in 1 study indicated a large effect size [23-25], 2 studies reported moderate effect sizes [15,26], and 1 study reported a small effect size [16]. In relation to the quality of the studies, 3 out of the 9 studies with positive outcomes [6,15,23-25] were within those classified as low risk of bias or those that had low risk of bias in 6 out of 7 categories, with the exception of blinding [6,23-25] (Table 2).

Of the 9 studies that demonstrated statistically significant improvement in pain, 1 was on participants with hip OA, 1 was on chronic knee and hip OA, 1 was on knee pain, and 5 were on LBP. Of the 10 studies that did not demonstrate improvement, 1 was on OA knee, 6 were on chronic LBP, 2 were on chronic musculoskeletal pain, and 1 was on work-related neck and upper limb disorders. The duration of the interventions varied from 3 weeks to 9 months in the studies that showed improvement and 3 weeks to 12 months in those studies that did not show positive outcomes.

### Functional Disability

In all, 16 of the included studies [6,14-16,18-27,29-33] used outcomes that assessed functional disability. Of these, 10 reported statistically significant improvements [6,15,16,20,21,23-27,29,32] (Multimedia Appendix 4); 5 of these studies [6,15,16,26,27] assessed outcomes at more than one time point, and in 2 studies [15,27], improvement was not maintained at the last time point measured. A total of 6 studies [15,16,20,21,23-26] reported effect sizes. In total, 3 studies reported a large effect size [15,16,23-25], 2 studies reported a moderate effect size [20,21], and 1 study reported a small effect size [26] (Multimedia Appendix 4). In relation to the quality of the studies, 5 out of the 10 studies with positive outcomes [6,15,21,23-25,27] had a low or relatively low risk of bias (Table 2).

Of the 10 studies that demonstrated statistically significant improvement in functional disability, 2 studies were on participants with chronic knee pain, 1 was on hip OA, 1 was on chronic knee and hip OA, 3 were on LBP, and 3 were on chronic LBP. Of the 6 studies that did not demonstrate improvement, 1 was on OA knee, 1 was on LBP, 2 were on chronic LBP, and 2 were on chronic musculoskeletal pain [14,18,19,22,30,31,33]. The duration of the interventions varied from 3 weeks to 9 months in the studies that showed improvement and 4 weeks to 4 months in those studies that did not show positive outcomes.

### Additional Outcome Measures

Several additional outcomes were measured across the studies including measures of quality of life, psychosocial distress, work, and surgery interest. Multimedia Appendix 2 gives details of all outcomes, and Multimedia Appendix 4 shows statistically significant results at the last measured time point for each study. The most frequent additional outcomes across the studies were catastrophizing, self-efficacy, quality of life, and coping strategies.

Of the 7 studies reporting on catastrophizing [6,15,17,20,22,31,33], 4 reported statistically significant improvements [6,15,17,20]. Moreover, 7 studies examined self-efficacy [6,15,16,18-20,22,31], 2 of which reported statistically significant improvements [6,20]. In all, 6 studies reported on health-related quality of life [6,15,17-19,23-26], of which 3 [6,17,23-25] reported statistically significant improvements. In total, 6 studies reported on coping ability [6,15,16,18,19,22,30], of which 4 [6,15,16,22] showed significant improvement. A total of 2 studies examined self-reported interest in surgery post intervention [29,32], and both the studies showed significant reductions in pursuing surgical intervention.

## Discussion

### Principal Findings

The primary aim of this systematic review was to assess if digital health interventions were clinically effective in impacting musculoskeletal pain and functional disability in patients with musculoskeletal conditions. The results of the analysis show that there is some evidence to support the effectiveness of digital health interventions in improving pain, with 9 out of 19 studies reporting significant improvements. There was stronger evidence to support the role of digital health interventions in improving functional disability, with 10 out of 16 studies reporting significant improvements. There were also positive results shown in several additional outcomes, most notably catastrophizing and coping strategies, with 4 out of 7 and 4 out of 6 studies, respectively, reporting significant improvements.

In terms of musculoskeletal conditions treated, both peripheral and spinal conditions showed improvement in pain and/or functional disability. However, pain outcomes in all studies with a study population of chronic musculoskeletal conditions (chronic low back and chronic musculoskeletal pain) did not show any significant improvements. This is not a surprising finding, as generally interventions for chronic low back and chronic musculoskeletal pain are likely to have a greater effect on function, quality of life, and psychosocial factors rather than pain [37], as was found in this review. The positive outcomes for the majority of studies that considered coping strategies and catastrophizing may indicate that digital education and management strategies enable patients to better understand and cope with their musculoskeletal condition. The reduction in interest in surgery found in 2 studies [29,32] supports this.

Overall, the methodological quality of the included trials was variable. Only 1 study [15] had a low risk of bias across all domains. In total, 6 further studies were unable to blind the study participants [6,14,21,23-25,27,31], which is a common challenge facing researchers developing pragmatic clinical trials with comparative interventions [36]. Therefore, the potential risk of performance bias is elevated [36]. However, there was no observed direct relationship between the quality of trials and positive or negative outcomes; 3 of the 7 studies with low or relatively low risk of bias demonstrated significant improvements in pain, and 5 studies demonstrated significant improvements in functional disability. The large number of outcome measures used by the included studies made direct comparisons between studies difficult. This is a common problem in musculoskeletal research and is a reflection of the large number of outcome measures used in musculoskeletal conditions. The digital health interventions also varied considerably in many aspects including duration, program features, and targeted musculoskeletal condition, meaning a meta-analysis was not possible.

The secondary aim of the study was to explore the content, characteristics, and delivery of digital health interventions to ascertain if there are specific aspects of the interventions, or the population they are targeting, that are associated with beneficial outcomes. In relation to this aim, we looked across all studies in an attempt to identify characteristics related to positive or negative outcomes. Several features emerged following the analysis of the studies.

It appears important to match the digital health intervention to known evidence-based approaches for the condition. Examples of this can be seen in the studies where the population had chronic LBP or chronic musculoskeletal pain. In 3 of the studies that did not show any significant improvements in functional disability [17,22,30], interventions were not matched to what would be considered the best evidence-based practice. It is widely recognized that chronic musculoskeletal conditions, particularly LBP, are optimally managed using a biopsychosocial approach [38] incorporating both physical and psychosocial elements in the rehabilitation program. Chiauzzi et al [22], Peters et al [30], and Buhrman et al [17] utilized components of a psychosocial approach within their digital health interventions, but they did not specify an exercise or physical activity component. None of these studies demonstrated improvement in pain or functional disability. In contrast, 3 studies [20,21,26] did achieve statistically significant improvements in functional disability when including an exercise/physical activity component alongside a psychosocial component within their digital health intervention. In total, 2 studies [18,19,31] demonstrated the inclusion of all components of a biopsychosocial approach but achieved no statistically significant improvement in functional disability; however, this can be explained to an extent by the nature of the studies. In Calner et al’s and Nordin et al’s studies [18,19], the control group received multimodal rehabilitation (MMR) treatment from a minimum of 3 different health care professionals, including physiotherapists, psychologists, physicians, occupational therapists, and nurses. The experimental group also received MMR plus a web-based behavior change program; therefore, both the experimental and control groups had access to extensive psychosocial and physical intervention. Petrozzi et al [31] conducted an established internet-delivered program designed for the prevention and management of depressive symptoms (MoodGYM) and conducted a single-blinded study to examine the effectiveness of this in combination with physical treatments for patients with chronic LBP. The lack of significant improvement may be a reflection of the mismatch of content to the target population. The population for this study had moderate levels of back pain, low levels of disability, high levels of self-efficacy, and normal to mild levels of psychological distress (as assessed by STarTBack screening tool). However, MoodGYM is targeted toward those with higher levels of psychological distress and at higher risk of ongoing disability. The authors themselves acknowledge this as a limitation of their study. This highlights the importance of content being appropriately targeted toward the intended audience.

Another feature we identified was that all the digital health interventions delivered on an app [21,26,29,32,33], as opposed to a web-based program, produced positive results in pain and/or functional disability. In the context of this review, apps appear to have gained popularity in recent years. Of the 5 studies using apps, 1 was published in 2015 and the remaining 4 in 2018-2019. A number of reasons can be hypothesized as to why apps may provide successful digital health interventions. All the apps had in-app functions that facilitated greater engagement with study participants, for example, sensor-guided exercise features, notifications, and daily activity goals. Additionally, the success of apps may be related to other factors, including ease of access, portability, and convenience in comparison to web-based interventions.

In several studies, additional efforts were made to encourage engagement with the digital intervention. Various forms of multimedia additional support were included in 10 studies, such as phone calls, email reminders, and text messages [6,14-17,23-26,29-31]. In total, 6 of these studies demonstrated positive results for either pain and/or functional disability. Therefore, there is some indication that these additional forms of support may be linked to positive outcomes; however, the frequency and delivery modes were variable; as such, it is difficult to quantify the extent to which additional forms of support improve the effectiveness of a digital health intervention.

Due to the different features within each intervention, it was difficult to draw any firm conclusions regarding which components of digital intervention create the most engaging digital interface. The number of participant-interactive components within web-based interventions (eg, exercise trackers, web-based coaching, and quizzes) did not appear to definitively influence the success of the intervention. Both significant and nonsignificant outcomes were seen in trials with multiple interactive elements (Multimedia Appendix 2). An RCT by Riva et al [39] was designed to specifically evaluate the addition of interactive features to a well-established internet intervention for chronic back pain; the results of this study showed no difference between the group with multiple interactive features and the control in relation to pain and physical activity. It is also unclear if tailoring the intervention offers additional benefits. One of the studies included in this review [26] was a 3-arm study comparing a control group, tailored mobile-web intervention, and an alternative care group that received emails directing participants to nontailored web-based resources. Significant reductions in function were reported at 16-week follow-up for both intervention groups compared with the control; however, there was no significant difference between the groups.

In all studies reviewed, there was minimal reference to patient involvement in the development of the digital health intervention. Many studies on such interventions appear to use content developed by the medical/research team, with little reference to patient involvement in the design phase of the intervention [40]. Involving patients early in the development process may help inform key features of the design, including what constitutes an engaging interface.

In 6 studies [6,15,18,19,29,31,33], digital health interventions were used as an adjunct to face-to-face intervention with a health care practitioner, that is, physiotherapist or multidisciplinary team; 4 of these studies [6,15,29,33] showed improvements in pain and/or functional disability. In the 2 studies in which digital intervention did not show any improvement [18,19,31], the studies by Calner et al and Nordin et al [18,19] involved extensive MDT rehabilitation in both groups, and the addition of web-based intervention did not improve outcomes. This may reflect the intensity of face-to-face treatment received by the study participants in both groups. Petrozzi et al’s study [31] was targeted at patients with high levels of psychosocial distress that did not match the presentation of the patients in the study group; the mismatch of the intervention to the target group may have influenced the results. As such, there is some support that digital health interventions may improve outcomes as an adjunct to face-to-face treatment with a health care practitioner. Importantly, in no cases were digital health interventions inferior to an interventional control in relation to pain or functional disability, and no trials reported adverse events. This, in itself, is an important finding, as although not always superior to interventional controls (usual care), digital health interventions have the ability to deliver safe, high-reach, low-cost, readily accessible, and scalable care. They could also help address physical access issues, as a result of the nature of a patient’s pain, comorbidities, travel distances, and costs. Therefore, the use of digital interventions as an alternative to usual care may have a substantial impact on helping to manage the growing burden of musculoskeletal functional disability. This is particularly pertinent in health care systems currently stretched to such an extent that the frequency of delivery of face-to-face appointments is suboptimal. Digital health interventions may ultimately result in patients accessing more health care than they would in a solely face-to-face scenario.

From the review, no conclusions can be drawn regarding the duration of intervention for both pain and functional disability. Significant and nonsignificant outcomes were found in both short duration (3 weeks) and longer duration studies (up to 12 months). Due to the heterogeneity of the interventions and the lack of detail in the studies, it is also difficult to draw conclusions on the optimal dose or exposure to digital health interventions required to gain meaningful benefits. Many studies did not quantify how long patients engaged with the digital intervention; therefore, it is not possible to conclude if patients who engaged for longer durations with the intervention did better or worse. Further research is needed in both areas.

In what is perhaps a reflection of the emerging role of digital health interventions in the management of musculoskeletal conditions, there was a lack of long-term follow-up, particularly for patients with chronic musculoskeletal conditions in the majority of studies. It would be beneficial to assess the impact of successful websites and apps over a longer duration, as this has potential implications for patients, services, and health care resources if acute exacerbations of chronic conditions can be, at least in part, managed remotely.

There are certain limitations to this systematic review. Only English language studies were included; therefore, it is possible that relevant literature published in other languages may have been excluded. In addition, this review only included RCT study designs; however, this was to ensure that higher levels of evidence were used to address the aim of the review [41]. Finally, it was not possible to undertake a meta-analysis due to the diversity across interventions and comparators in the reviewed trials [13].

### Conclusions

This review has demonstrated that digital health interventions have some clinical benefits in the management of musculoskeletal conditions. There is evidence to support the effectiveness of digital health interventions in improving pain. There is stronger evidence to support the effectiveness of digital health interventions in improving functional disability. There are also positive results shown in several additional outcomes, notably catastrophizing and coping strategies. This review demonstrates the potential of digital health interventions to contribute positively toward diminishing the personal, societal, and economic impact of musculoskeletal conditions, which, as our population ages, is only set to grow. Further research is needed to identify the patient subgroups that respond most positively to digital health interventions and also to determine the pertinent features of the interventions that are likely to achieve more successful patient outcomes.

## Appendix

Multimedia Appendix 1 Search terms.

Multimedia Appendix 2 Intervention characteristics.

Multimedia Appendix 3 Search strategies.

Multimedia Appendix 4 Results for pain and functional disability outcomes and for significant additional outcomes.

Multimedia Appendix 5 Risk of bias assessment.

Multimedia Appendix 6 Abbreviations for outcomes measures.

## Abbreviations

LBP: low back pain
MMR: multimodal rehabilitation
OA: osteoarthritis
PRISMA: Preferred Reporting Items for Systematic Reviews and Meta-Analyses
RCT: randomized controlled trial
