# Supplementary material for: The Effectiveness of Digital Health Interventions in the Management of Musculoskeletal Conditions: Systematic Literature Review
Source: J Med Internet Res. 2020 Jun 5;22(6):e15617. doi: 10.2196/15617 (PMC7305565; doi:10.2196/15617)
Supplement: Multimedia Appendix 1 [file jmir_v22i6e15617_app1.docx]

**Multimedia Appendix 1 – Search terms**

| **Population:**  Musculoskeletal Conditions | Back pain; Neck pain; Spin* pain; Thoracic pain; Cervical pain; Lumbar pain; Back ache; Low back pain; Knee pain; Shoulder pain; Elbow pain; Wrist pain; Hand pain; Ankle pain; Foot pain; Hip pain; Musculoskeletal |
| --- | --- |
| **Intervention:**  Digital interventions | Smartphone/smart phone; Internet; Software; Mobile application; Software design; Website; Web site; Webpage; Web page; Computer based; Computer assisted; Digital; Online; App; Apps |
| **Study Design** | Controlled clinical trial; Randomised controlled trial; Clinical trial; Metaanalys* OR meta analys* OR meta-analys*; Systematic review; Random*; Trial*; Randomis* OR Randomiz*; Random* ADJ2 trial*; Randomly ADJ2 (allocated OR assigned); (Single OR double OR triple OR treble) AND (blind* OR mask*) |
