# Supplementary material for: The Effectiveness of Digital Health Interventions in the Management of Musculoskeletal Conditions: Systematic Literature Review
Source: J Med Internet Res. 2020 Jun 5;22(6):e15617. doi: 10.2196/15617 (PMC7305565; doi:10.2196/15617)
Supplement: Multimedia Appendix 2 [file jmir_v22i6e15617_app2.docx]

**Multimedia Appendix 2.** Intervention characteristics.

| Authors, ~~year,~~ and country of origin | Mode of delivery | Recommended frequency and intervention duration | Interactive element | Control | Outcome measures |
| --- | --- | --- | --- | --- | --- |
| Allen et al [21], USA | EG^a^ 1: Face-to-face physiotherapy  EG2: Web-based | EG1: 8×1-hour sessions within 4 months  EG2: complete strengthening and stretching exercises at least three times per week and to engage in aerobic exercises daily  Duration=12 months | EG1: physiotherapy including assessment, assistive devices, home exercises, activity pacing, manual therapy, and pain management as appropriate.  EG2: IBET^b^ program include tailored exercises, exercise progression, video display and photographs of exercises, automated reminders, and progress tracking | Waiting list control | - *Western Ontario and McMaster Universities Osteoarthritis Index* - Physical function as measured by 30-second chair stand - Timed Up and Go - 2-min step test - Unilateral stand time - Physical Activity Scale for the Elderly |
| Bennell et al [6], Australia | Web-based | Both groups: Access educational material at leisure.  EG: Complete 8 35-45 min modules (1 per week, starting week 1). Practice pain coping skills daily over 12 weeks  Seven 30-45 min Skype sessions with a physiotherapist over 12 weeks  Continue on Web education until 9 months | Internet-based educational material  PCST^c^ training for 3 months (PainCOACH). Weekly email for 8 weeks to remind participants to complete weekly module. After week 8, received monthly reminder  Skype sessions with physiotherapy for home exercise program | Internet-based educational material | - *Numeric Rating Scale (pain during walking)* - *Physical functioning (WOMAC^d^)* - Knee pain (WOMAC) - Assessment of Quality of Life -2 - Arthritis Self-Efficacy Scale - Pain Catastrophising Scale - Coping Strategies Questionnaire - Global change (overall and pain) |
| Bennell et al [29], Australia | Web-based and face-to-face physiotherapy in both groups | Both groups: Attended 5 face-to-face 30 min 1:1 sessions with the physiotherapist for 8-24 weeks  To continue with exercise program until 52 weeks—3 times/week  EG: + Eight 35 to 45-minute modules. One module/week for 8 weeks. | EG: In the first 8 weeks received Web-based education as CG^e^ + internet-delivered PCST program. Weekly email for 8 weeks to remind participants to complete weekly module. After week 8, received monthly reminder  From weeks 8 to 24, both groups visited a physiotherapist 5 times for home exercise prescription. | CG—In the first 8 weeks received Web-based education only including on osteoarthritis, pain, physical activity, and emotions.  From weeks 8 to 24, both groups visited a physiotherapist 5 times for home exercise prescription. | - *Numeric Rating Scale* - *Western Ontario and McMaster Universities Osteoarthritis Index* - Assessment of Quality of Life - Arthritis Self-Efficacy Scale - Coping Strategies Questionnaire - Pain Catastrophising Scale - Depression Anxiety Stress Scale - Physical Activity Scale for the Elderly - Program adherence |
| Bossen et al [33], Netherlands | EG: Web-based | 9-week program.  1 module to be completed each week. | Join2move including goal setting, Web-based text messages, and emails to promote physical activity | Waiting list control | - *Physical Activity Scale for the Elderly total Physical Activity (accelerometer)* - *Physical functioning (Hip Injury and Osteoarthritis Outcome Score and Knee Injury and Osteoarthritis Outcome Score)* - *Self-perceived* *effect* - Sedentary intensity, pain, tiredness, symptoms, quality of life, sport/recreation/self-efficacy pain, self-efficacy, and other symptoms |
| Buhrman et al [34], Sweden | EG: Web-based | Weekly modules with weekly assignments for 12 weeks | Self-help management program based on CBT^f^ including education, cognitive skills acquisition, and maintenance. Plus, a telephone call by a therapist to give the opportunity to ask questions. Reminders were sent to participants when reports on progress were not delivered as expected. | Waiting list control | - *Coping Strategies Questionnaire* - Multidimensional Pain Inventory - Pain and Impairment Relationship Scale - Hospital Anxiety and Depression Scale - Quality of Life Inventory |
| Calner et al [19], and Nordin et al [20], Sweden | CG: Face-to-face  EG: Web-based | CG: a minimum of 2-3 treatment sessions/week for at least 6 weeks  EG: Access was restricted to 1 new module per week during the first 8 weeks. Access for 16 weeks | EG: MMR (multimodal pain rehabilitation) + Web-BCPA (Web behavior change program for activity).  The Web-BCPA focus on function and activity to increase the participants’ physical and cognitive activity in their rehabilitation. Web-BCPA consisted of 8 modules which contained information, assignments, and exercises. | CG: MMR (multimodal pain rehabilitation)  Treatment from a minimum of 3 different health care professionals including physiotherapist, physician, occupational therapist, psychologist, and nurse, working to CBT approach. | - *Work Ability Index* - *Working %* - Pain Visual Analogue Scale - Pain Disability Index - Short form Medical Outcomes Study 36 - Web Behaviour Change Program for Activity adherence - Satisfaction - Self-efficacy: Arthritis Self-Efficacy Scale;   General Self-Efficacy Questionnaire   - Coping Strategies Questionnaire |
| Carpenter et al [22], USA | Web-based | Twice/week, email reminders.  3-week intervention period | Web-based CBT intervention (WW^g^). Reflective and interactive exercises | Waiting list control for 3 weeks, then given access to website and WW | - *Survey of Pain Attitudes* - Fear Avoidance Belief Questionnaire - Negative Mood Regulation Scale - Pain Catastrophising Scale - Roland Morris Disability Questionnaire - Numeric Rating Scale |
| Chhabra et al [36], India | Snapcare app | Daily activity goals for 12 weeks | Written prescription as CG + Snapcare app, which included daily activity goals, notifications, and surveys | Written prescription from the physician, which included medicines and the recommended level of physical activity | - *Pain Numeric Rating Scale* - *Modified Oxford Disability Index* - Current Symptom Score |
| Chiauzzi et al [23], USA | Web-based | CG: to read back pain guide over a 4-week period.  EG: 2 times/week for 4 weeks=8 sessions total, then unlimited for 6 months | Interactive self-management website based on CBT. Web-based content reviewed with instructions for first 4 weeks. Plus 5 monthly follow-up reviews | Information only: Emailed a back pain guide | - *Brief Pain Inventory* - *Oswestry Disability Questionnaire* - *Depression Anxiety Stress Scale* - *Participants’ Global Impression of Change* - Chronic Pain Coping Inventory-42 - Pain Catastrophising Scale - Pain Self-Efficacy Questionnaire - Fear Avoidance Belief Questionnaire |
| Del Pozo-Cruz et al [16-18], Spain | EG: Web-based  Plus, both groups ×1 face-to-face visit | EG: engage in Web-based program for 11 min each day, 5 days a week for 9 months | Standard care + Web-based postural and exercise intervention Web-based program with personal daily emails | Standard care: self-care Web-based information based including ergonomic material, plus at least one visit once per year | - *Roland Morris Disability Questionnaire* - STarTBack Screening Tool - EuroQol – Five Dimensions – Three Levels Health Questionnaire - Oswestry Disability Questionnaire |
| Irvine et al [24], USA | EG1: FitBack app  EG2: Web-based links and emails | Unlimited access. Weekly reminders to visit app  8-week intervention period, access for 16 weeks | EG1: FitBack intervention based on CBT.  Provides low back pain education and management strategies for current episodes and prevention plans for future episodes. Weekly email reminders for 8 weeks; emails to complete assessments  EG2=Alternative care group: participants received 8 emails with links to websites | Usual care, emails to request completion of questionnaire | - Multidimensional Pain Inventory - Dartmouth Co-operative Functional Assessment Chart - Work Limitation Questionnaire - Stanford Presenteeism Scale - Patient Activation Measure - Theory of Planned Behaviour Constructs |
| Krein et al [25], USA | Web-based | Unlimited access with weekly reminders  12-month intervention period | Internet-mediated intervention including pedometer, feedback, goal setting, targeted messages, and Web-based e-community | Usual care including attending Back Class and pedometer.  Did not receive any feedback or have access to the website. Email reminders | - *Roland Morris Disability Questionnaire* - Short form Medical Outcomes Study 36 - Short form Medical Outcomes Study 36 - Fear Avoidance Belief Questionnaire - Exercise regularly scale |
| Marangoni [26], USA | Web-based | Both EG groups to stretch once every 6 min for 15-17 work days | EG1: Stretching exercises using CASP^h^. Reminded to stretch via CASP every 6 min while the computer was operating  EG2: Stretching exercises using FLIP^i^—hard copy of the CASP program | No intervention | - *Pain intensity Visual Analogue Scale* - *Pain Spot Assessment* |
| Mecklenberg et al [27], USA | EG: Hinge Health app | EG: 3 sessions exercise therapy, reading 1-2 papers, performing CBT, at least three 30-min sessions of aerobic activities per week for 12 weeks | *Hinge Health* 12-week digital care package for chronic knee pain, personal coach via app, text/email message reminders | 3 pieces of education presented digitally | - *Knee Injury and Osteoarthritis Outcome Score pain subscale* - *Knee Injury and Osteoarthritis Outcome Score Physical Function Short form* - Visual Analogue Scale pain - Visual Analogue Scale stiffness - Surgery chance next year %; surgery chance next 2 years %; surgery chance next 5 years; surgery interest; understanding |
| Peters et al [31], Netherlands | Web-based | EG1 and EG2 consisted of 8 modules.  One new module per week for 8 weeks.  All modules available at 8 weeks. | EG1 iCBT: internet-delivered cognitive behavioral program  EG2 positive psychology intervention self-help program called “Happy Despite Pain.” Included 4 positive psychology exercises.  Both groups received telephone and email support | Waitlist | - *Hospital Anxiety and Depression Scale* - *Happiness (single-item question)* - *Fibromyalgia Impact Questionnaire* - Pain Visual Analogue Scale - Self-Compassion Scale - Mood and optimism - Pain Catastrophising Scale - Illness coping |
| Petrozzi et al [30], Australia | CG: Face-to-face sessions  EG: Face-to-face sessions + website access to MoodGYM | CG: Up to 12 sessions—frequency and number of sessions determined by clinical judgment.  EG: As CG + completion of 5 modules; 1 per week while undertaking their physical treatments  Intervention for a maximum of 8 weeks | Physical treatments + MoodGYM program. MoodGYM used for teaching people to better respond to psychological distress. Written information, quizzes, within CBT framework. Weekly telephone call to assess and encourage adherence to MoodGYM. | Physical treatments by a chiropractor or physiotherapist. Included manual therapy, advice, education, and exercise | - *Pain Self-Efficacy Questionnaire* - *Roland Morris Disability Questionnaire* - *Pain* Catastrophising Scale - Patient Specific Functional Scale - Depression Anxiety Stress Scale 21 - Pain Numeric Rating Scale - Work Ability Index |
| Shebib et al [28], USA | App | EG: 3 sessions exercise therapy, reading 1-2 papers, performing CBT, at least three 30-min sessions of aerobic activities per week for 12 weeks | 12-week digital care program—exercise therapy, education, CBT, behavioral coaching, activity tracker, symptoms tracker | 3 × digital educational papers only | - *Oswestry Disability Index* - *Modified Von Korff scale (Pain)* - *Modified Von Korff scale (Disability)* - Understanding of Low Back Pain and back surgery interest |
| Toelle et al [35], Germany | CG: 1:1 face-to-face sessions + links to websites  EG: Kaia app | CG: once per week for 6 weeks  EG: 4 times a week for 3 months | Multidisciplinary mhealth^j^ back pain app—3 modules: (1) education on back pain, (2) exercise, and (3) mindfulness and relaxation techniques | 6 individual physiotherapy sessions including manual therapy, exercise and advice + 6 emails with links to Web-based education on various issues related to back pain including pathology and self-management and motivational messages. | - *Pain intensity Numeric Rating Scale* - Hanover functional ability questionnaire - Veterans RAND 12-item health survey - Graded chronic pain scale - Mental component summary score - Pain Component Summary Score |
| Van den Heuvel et al [32], Netherlands | Web-based | EG1: On screen signal to take rest break for 5 min after 35 min continuous computer usage. Microbreak of 7 seconds after 5 min of continuous computer usage for 8 weeks  EG2: breaks as above + 4 exercises at the start of each break for 8 weeks | EG1: computer program to stimulate regular breaks among computer users  EG2=breaks as above + exercises | No intervention | - *Perceived* *overall recovery from complaint (7-point scale)* - Frequency and severity of complaints; self- reported sick leave; mean number key strokes/day |

^a^EG: experimental group.

^b^IBET: internet-based exercise training.

^c^WOMAC – Western Ontario and McMaster Universities Osteoarthritis Index

^d^PCST: pain coping skills training.

^e^CG: control group.

^f^CBT: cognitive behavioral therapy.

^g^WW: wellness workbook.

^h^CASP: computer-assisted stretching program.

^i^FLIP: facsimile lesson with instructional pictures.

^j^mhealth: mobile health.

Italic outcome measures indicate the paper’s primary outcome measure, where identified.

References:

[6] Bennell KL, Nelligan R, Dobson F, Rini C, Keefe F, Kasza J, French S, Bryant C, Dalwood A, Abbott JH, Hinman RS. Effectiveness of an internet-delivered exercise and pain-coping skills training intervention for persons with chronic knee pain: a randomized trial. Ann Intern Med 2017 Apr 4; 166(7):453-62

[16] del Pozo-Cruz B, Parraca JA, del Pozo-Cruz J, Adsuar JC, Hill J, Gusi N. An occupational, internet-based intervention to prevent chronicity in subacute lower back pain: a randomised controlled trial. J Rehabil Med 2012 Jun; 44(7):581-7

[17] del Pozo-Cruz B, Gusi N, del Pozo-Cruz J, Adsuar JC, Hernandez-Mocholí M, Parraca JA. Clinical effects of a nine-month web-based intervention in subacute non-specific low back pain patients: a randomized controlled trial. Clin Rehabil 2013 Jan; 27(1):28-39

[18] del Pozo-Cruz B, Adsuar JC, Parraca J, del Pozo-Cruz J, Moreno A, Gusi N. A web-based intervention to improve and prevent low back pain among office workers: a randomized controlled trial. J Orthop Sports Phys Ther 2012 Oct; 42(10):831-41

[19] Calner T, Nordin C, Eriksson MK, Nyberg L, Gard G, Michaelson P. Effects of a self-guided, web-based activity programme for patients with persistent musculoskeletal pain in primary healthcare: a randomized controlled trial. Eur J Pain 2017 Jul; 21(6):1110-20

[20] Nordin CA, Michaelson P, Gard G, Eriksson MK. Effects of the web behavior change program for activity and multimodal pain rehabilitation: randomized controlled trial. J Med Internet Res 2016 Oct 5; 18(10):e265

[21] Allen KD, Arbeeva L, Callahan LF, Golightly YM, Goode AP, Heiderscheit BC, Huffman KM, Severson HH, Schwartz TA. Physical therapy vs internet-based exercise training for patients with knee osteoarthritis: results of a randomized controlled trial. Osteoarthritis Cartilage 2018 Mar; 26(3):383-96

[22] Carpenter KM, Stoner SA, Mundt JM, Stoelb B. An online self-help CBT intervention for chronic lower back pain. Clin J Pain 2012 Jan; 28(1):14-22

[23] Chiauzzi E, Pujol LA, Wood M, Bond K, Black R, Yiu E, Zacharoff K. painACTION-back pain: a self-management website for people with chronic back pain. Pain Med 2010 Jul; 11(7):1044-58

[24] Irvine AB, Russell H, Manocchia M, Mino DE, Glassen TC, Morgan R, Gau JM, Birney AJ, Ary DV. Mobile-web app to self-manage low back pain: randomized controlled trial. J Med Internet Res 2015 Jan 2; 17(1):e1

[25] Krein SL, Kadri R, Hughes M, Kerr EA, Piette JD, Holleman R, Kim HM, Richardson CR. Pedometer-based internet-mediated intervention for adults with chronic low back pain: randomized controlled trial. J Med Internet Res 2013 Aug 19; 15(8):e181

[26] Marangoni AH. Effects of intermittent stretching exercises at work on musculoskeletal pain associated with the use of a personal computer and the influence of media on outcomes. Work 2010; 36(1):27-37

[27] Mecklenburg G, Smittenaar P, Erhart-Hledik JC, Perez DA, Hunter S. Effects of a 12-week digital care program for chronic knee pain on pain, mobility, and surgery risk: randomized controlled trial. J Med Internet Res 2018 Apr 25; 20(4):e156

[28] Shebib R, Bailey JF, Smittenaar P, Perez DA, Mecklenburg G, Hunter S. Randomized controlled trial of a 12-week digital care program in improving low back pain. NPJ Digit Med 2019; 2:1

[29] Bennell KL, Nelligan RK, Rini C, Keefe FJ, Kasza J, French S, Forbes A, Dobson F, Abbott JH, Dalwood A, Harris A, Vicenzino B, Hodges PW, Hinman RS. Effects of internet-based pain coping skills training before home exercise for individuals with hip osteoarthritis (HOPE trial): a randomised controlled trial. Pain 2018 Sept; 159(9):1833-42

[30] Petrozzi MJ, Leaver A, Ferreira PH, Rubinstein SM, Jones MK, Mackey MG. Addition of MoodGYM to physical treatments for chronic low back pain: a randomized controlled trial. Chiropr Man Therap 2019; 27:54

[31] Peters ML, Smeets E, Feijge M, van Breukelen G, Andersson G, Buhrman M, Linton SJ. Happy despite pain: a randomized controlled trial of an 8-week internet-delivered positive psychology intervention for enhancing well-being in patients with chronic pain. Clin J Pain 2017 Nov; 33(11):962-75

[32] van den Heuvel SG, de Looze MP, Hildebrandt VH, Thé KH. Effects of software programs stimulating regular breaks and exercises on work-related neck and upper-limb disorders. Scand J Work Environ Health 2003 Apr; 29(2):106-16

[33] Bossen D, Veenhof C, van Beek KE, Spreeuwenberg PM, Dekker J, de Bakker DH. Effectiveness of a web-based physical activity intervention in patients with knee and/or hip osteoarthritis: randomized controlled trial. J Med Internet Res 2013 Nov 22; 15(11):e257

[34] Buhrman M, Nilsson-Ihrfeldt E, Jannert M, Ström L, Andersson G. Guided internet-based cognitive behavioural treatment for chronic back pain reduces pain catastrophizing: a randomized controlled trial. J Rehabil Med 2011 May; 43(6):500-5

[35] Toelle T, Utpadel-Fischler D, Haas K, Priebe J. App-based multidisciplinary back pain treatment versus combined physiotherapy plus online education: a randomized controlled trial. NPJ Digit Med 2019; 2:34

[36] Chhabra HS, Sharma S, Verma S. Smartphone app in self-management of chronic low back pain: a randomized controlled trial. Eur Spine J 2018 Nov; 27(11):2862-74
