# Supplementary material for: The Effectiveness of Digital Health Interventions in the Management of Musculoskeletal Conditions: Systematic Literature Review
Source: J Med Internet Res. 2020 Jun 5;22(6):e15617. doi: 10.2196/15617 (PMC7305565; doi:10.2196/15617)
Supplement: Multimedia Appendix 3 [file jmir_v22i6e15617_app3.docx]

**Multimedia Appendix 3**. Search Strategies

| Database | Hits |
| --- | --- |
| MEDLINE | 205 |
| CINAHL | 420 |
| Embase | 186 |
| SCOPUS | 236 |
| Total | 1047 |

The initial search was conducted on 28/02/2019. It was then repeated on 15/11/2019. The search of the MEDLINE database has been provided as an example of the search strategy used:

01/01/2000 – 28/02/2019 (initial search)

Repeat searches 28/02/2019 – 15/11/2019

| 87 | Medline | SMARTPHONE/ OR INTERNET/ OR "MOBILE APPLICATIONS"/ OR SOFTWARE/ OR "SOFTWARE DESIGN"/ | 156097 |
| --- | --- | --- | --- |
| 88 | Medline | (website*).ti,ab | 19856 |
| 89 | Medline | (webpage*).ti,ab | 495 |
| 90 | Medline | ("web page*").ti,ab | 1567 |
| 91 | Medline | ("web site*").ti,ab | 7213 |
| 92 | Medline | ("computer based*").ti,ab | 12330 |
| 93 | Medline | ("computer assisted*").ti,ab | 22716 |
| 94 | Medline | (digital).ti,ab | 99774 |
| 95 | Medline | (online).ti,ab | 84392 |
| 96 | Medline | ("mobile application*").ti,ab | 1150 |
| 97 | Medline | (app OR apps).ti,ab | 20257 |
| 98 | Medline | (internet OR smartphone* OR "smart phone*").ti,ab | 52150 |
| 99 | Medline | (87 OR 88 OR 89 OR 90 OR 91 OR 92 OR 93 OR 94 OR 95 OR 96 OR 97 OR 98) | 437584 |
| 100 | Medline | ("back pain").ti,ab | 39150 |
| 101 | Medline | ("neck pain").ti,ab | 7720 |
| 102 | Medline | ("spin* pain").ti,ab | 1555 |
| 103 | Medline | ("thoracic pain").ti,ab | 1080 |
| 104 | Medline | ("cervical pain").ti,ab | 865 |
| 105 | Medline | ("lumbar pain").ti,ab | 1312 |
| 106 | Medline | "BACK PAIN"/ OR "LOW BACK PAIN"/ | 34397 |
| 107 | Medline | "NECK PAIN"/ | 5886 |
| 108 | Medline | KNEE/ AND pain | 1399 |
| 109 | Medline | ("knee pain").ti,ab | 5983 |
| 110 | Medline | "SHOULDER PAIN"/ | 3980 |
| 111 | Medline | ("shoulder pain").ti,ab | 5475 |
| 112 | Medline | ELBOW/ AND pain | 516 |
| 113 | Medline | ("elbow pain").ti,ab | 585 |
| 114 | Medline | (HAND/ OR WRIST/) AND pain | 3091 |
| 115 | Medline | ("wrist pain").ti,ab | 1206 |
| 116 | Medline | ("hand pain").ti,ab | 454 |
| 117 | Medline | (FOOT/ OR ANKLE/) AND pain | 3060 |
| 118 | Medline | ("ankle pain").ti,ab | 844 |
| 119 | Medline | ("foot pain").ti,ab | 1067 |
| 120 | Medline | HIP/ AND pain | 1105 |
| 121 | Medline | ("hip pain").ti,ab | 2888 |
| 122 | Medline | exp "MUSCULOSKELETAL PAIN"/ OR "musculoskeletal pain" | 8182 |
| 123 | Medline | (100 OR 101 OR 102 OR 103 OR 104 OR 105 OR 106 OR 107 OR 108 OR 109 OR 110 OR 111 OR 112 OR 113 OR 114 OR 115 OR 116 OR 117 OR 118 OR 119 OR 120 OR 121 OR 122) | 103009 |
| 125 | Medline | "RANDOMIZED CONTROLLED TRIALS AS TOPIC"/ | 113874 |
| 126 | Medline | "CONTROLLED CLINICAL TRIALS AS TOPIC"/ | 5301 |
| 127 | Medline | "CLINICAL TRIALS AS TOPIC"/ | 182469 |
| 128 | Medline | "META-ANALYSIS AS TOPIC"/ | 15951 |
| 129 | Medline | (random* ADJ2 trial*).ti | 102864 |
| 130 | Medline | (randomis* OR randomiz*).ti,ab | 525520 |
| 131 | Medline | (randomly ADJ2 (allocated OR assigned)).ti,ab | 118267 |
| 132 | Medline | ((single OR double OR triple OR treble) AND (blind* OR mask*)).ti,ab | 170322 |
| 133 | Medline | (metaanalys* OR "meta analys*" OR meta-analys*).ti | 72902 |
| 134 | Medline | ("systematic review*").ti | 79695 |
| 135 | Medline | (125 OR 126 OR 127 OR 128 OR 129 OR 130 OR 131 OR 132 OR 133 OR 134) | 943929 |
| 136 | Medline | (99 AND 123 AND 135) | 293 |
| 137 | Medline | 136 [DT FROM 2000] [Languages English] | 278 |
| 138 | Medline | (99 AND 123) | 1552 |
| 139 | Medline | 138 [DT FROM 2000] [Document type Clinical Trial OR Controlled Clinical Trial OR Meta-analysis OR Randomized Controlled Trial] [Languages English] | 200 |

Repeat searches 28/02/2019 – 15/11/2019

| 88 | Medline | (website*).ti,ab | 19856 |
| --- | --- | --- | --- |
| 89 | Medline | (webpage*).ti,ab | 495 |
| 90 | Medline | ("web page*").ti,ab | 1567 |
| 91 | Medline | ("web site*").ti,ab | 7213 |
| 92 | Medline | ("computer based*").ti,ab | 12330 |
| 93 | Medline | ("computer assisted*").ti,ab | 22716 |
| 94 | Medline | (digital).ti,ab | 99774 |
| 95 | Medline | (online).ti,ab | 84392 |
| 96 | Medline | ("mobile application*").ti,ab | 1150 |
| 97 | Medline | (app OR apps).ti,ab | 20257 |
| 98 | Medline | (internet OR "smart phone*" OR smartphone*).ti,ab | 52056 |
| 99 | Medline | (87 OR 88 OR 89 OR 90 OR 91 OR 92 OR 93 OR 94 OR 95 OR 96 OR 97 OR 98) | 436950 |
| 100 | Medline | ("back pain").ti,ab | 39150 |
| 101 | Medline | ("neck pain").ti,ab | 7720 |
| 102 | Medline | ("spin* pain").ti,ab | 1555 |
| 103 | Medline | ("thoracic pain").ti,ab | 1080 |
| 104 | Medline | ("cervical pain").ti,ab | 865 |
| 105 | Medline | ("lumbar pain").ti,ab | 1312 |
| 106 | Medline | "BACK PAIN"/ OR "LOW BACK PAIN"/ | 34397 |
| 107 | Medline | "NECK PAIN"/ | 5886 |
| 108 | Medline | KNEE/ AND pain | 1399 |
| 109 | Medline | ("knee pain").ti,ab | 5983 |
| 110 | Medline | "SHOULDER PAIN"/ | 3980 |
| 111 | Medline | ("shoulder pain").ti,ab | 5475 |
| 112 | Medline | ELBOW/ AND pain | 516 |
| 113 | Medline | ("elbow pain").ti,ab | 585 |
| 114 | Medline | (HAND/ OR WRIST/) AND pain | 3091 |
| 115 | Medline | ("wrist pain").ti,ab | 1206 |
| 116 | Medline | ("hand pain").ti,ab | 454 |
| 117 | Medline | (FOOT/ OR ANKLE/) AND pain | 3060 |
| 118 | Medline | ("ankle pain").ti,ab | 844 |
| 119 | Medline | ("foot pain").ti,ab | 1067 |
| 120 | Medline | HIP/ AND pain | 1105 |
| 121 | Medline | ("hip pain").ti,ab | 2888 |
| 122 | Medline | exp "MUSCULOSKELETAL PAIN"/ | 3305 |
| 123 | Medline | (100 OR 101 OR 102 OR 103 OR 104 OR 105 OR 106 OR 107 OR 108 OR 109 OR 110 OR 111 OR 112 OR 113 OR 114 OR 115 OR 116 OR 117 OR 118 OR 119 OR 120 OR 121 OR 122) | 92981 |
| 125 | Medline | "RANDOMIZED CONTROLLED TRIALS AS TOPIC"/ | 113874 |
| 126 | Medline | "CONTROLLED CLINICAL TRIALS AS TOPIC"/ | 5301 |
| 127 | Medline | "CLINICAL TRIALS AS TOPIC"/ | 182469 |
| 128 | Medline | "META-ANALYSIS AS TOPIC"/ | 15951 |
| 129 | Medline | (random* ADJ2 trial*).ti | 102864 |
| 130 | Medline | (randomis* OR randomiz*).ti,ab | 525520 |
| 131 | Medline | (randomly ADJ2 (allocated OR assigned)).ti,ab | 118267 |
| 132 | Medline | ((single OR double OR triple OR treble) AND (blind* OR mask*)).ti,ab | 170322 |
| 133 | Medline | (metaanalys* OR "meta analys*" OR meta-analys*).ti | 72902 |
| 134 | Medline | ("systematic review*").ti | 79695 |
| 135 | Medline | (125 OR 126 OR 127 OR 128 OR 129 OR 130 OR 131 OR 132 OR 133 OR 134) | 943929 |
| 136 | Medline | (99 AND 123 AND 135) | 271 |
| 137 | Medline | 136 [DT FROM 2000] [Languages English] | 256 |
| 138 | Medline | (99 AND 123) | 1546 |
| 139 | Medline | 138 [DT FROM 2000] [Document type Clinical Trial OR Controlled Clinical Trial OR Meta-analysis OR Randomized Controlled Trial] [Languages English] | 192 |
| 192 | Medline | 138 [DT 2019-2019] [Document type Clinical Trial OR Controlled Clinical Trial OR Meta-analysis OR Randomized Controlled Trial] [Languages English] | 5 |
