# Supplementary material for: The Effectiveness of Digital Health Interventions in the Management of Musculoskeletal Conditions: Systematic Literature Review
Source: J Med Internet Res. 2020 Jun 5;22(6):e15617. doi: 10.2196/15617 (PMC7305565; doi:10.2196/15617)
Supplement: Multimedia Appendix 4 [file jmir_v22i6e15617_app4.docx]

**Multimedia Appendix 4.** Results for pain and functional disability outcomes, and for significant additional outcomes

| Authors | Pain | Functional disability | Additional outcomes^a,b^ |
| --- | --- | --- | --- |
| Allen et al [21] | No between-group differences for EG^c^1 (PT – physical therapy) versus CG, EG2 (IBET—Web-based) versus CG^d^, and EG1 versus EG2 in WOMAC^e^ (pain) at 4 months or 12 months | No between-group differences for EG1 versus CG, EG2 versus CG, or EG1 versus EG2 in WOMAC (function) at 4 months or 12 months | No between-group differences for EG1 versus EG2 for all other measures at any time point |
| Bennell et al [6] | Significant improvement in pain NRS^f^ during walking for EG at 3 months (between-group mean difference 1.6; 95% CI 0.9 to 2.3; *P*<.001) and 9 months (between- group mean difference 1.1; 95% CI 0.4 to 1.8; *P*=.003)  Significant improvement in WOMAC (pain) in EG at 3 months (between-group mean difference 2.5; 95% CI 1.5 to 3.5; *P*<.001) and 9 months (between-group mean difference 1.6; 95% CI 0.6 to 2.6; *P*=.003) | Significant improvement in WOMAC (physical functioning) for EG at 3 months (*P*<.001; between-group mean difference 9.3; 95% CI 5.9 to 12.7; *P*<.001) and 9 months (between-group mean difference 7.0; 95% CI 3.4 to 10.5; *P*<.001) | Significant improvement in Assessment of Quality of Life for EG at 9 months (between-group mean difference −0.1; 95% CI −0.1 to 0; *P*=.02 ~~018~~), Pain Catastrophising Scale (between-group mean difference 0.5; 95% CI 0-1.0; *P*=.049), Coping Strategies Questionnaire (between-group mean difference −11.6; 95% CI −18.7 to −4.4; *P*=.002), Arthritis Self-Efficacy Scale pain (between-group mean difference −1.2; 95% CI −1.9 to −0.6; *P*<.001) |
| Bennell et al [29] | No between-group differences in NRS at 8, 24 or 52 weeks  Significant improvement in global pain rating for EG at 8 weeks (odds ratio [OR] 3.31, 95% CI 1.02 to 10.78; *P*=.04), no between-group difference at 24 or 52 weeks | Significant difference in WOMAC function at 8 weeks for EG (between-group mean difference −3.2; 95% CI −6.2 to −0.1; *P*=.04~~1~~), no between-group difference at 24 or 52 weeks  Significant improvement in global function rating for EG at 8 weeks (OR 6.93, 95% CI 1.75 to 27.49; *P*=.009), no between-group difference at 24 or 52 weeks | Significant improvement in Coping Strategies Questionnaire for EG at 52 weeks (between-group mean difference 15.3; 95% CI 4.4 to 26.2; *P*=.009) |
| Bossen et al [33] | Significant improvement in pain NRS for EG at 3 months (between-group mean difference −1.0; 95% CI −1.6 to −0.38; *P*=.002; Cohen *d*=−0.2)  No between-group difference in pain NRS at 12 months | Significant improvement in physical functioning subscale of KOOS^g^ and HOOS^h^ for EG at 3 months (between-group mean difference 6.5; 95% CI 1.8 to 11.2; *P*=.06; Cohen *d*=0.2) and self-perceived effect (OR 10.7, 95% CI 4.3 to 26.4; *P*<.001)  No between-group differences in physical activity as measured with Physical Activity Scale for the Elderly at 3 months  Significant improvement in total physical activity Physical Activity Scale for the Elderly for EG at 12 months (between-group mean difference 21.2, 95% CI 3.6 to 38.9; *P*=.02; Cohen *d*=0.18) and accelerometer min/day (between-group mean difference 24; 95% CI 0.5 to 46.8; Cohen *d*=0.19; *P*=.045) | Significant improvement in tiredness for EG at 12 months (between-group mean difference 1.15; 95% CI −1.9 to −0.28; *P*=.008; Cohen *d*=−0.22); passive pain coping scores (between-group mean difference −0.12; 95% CI −0.2 to −0.03; *P*=.008; Cohen *d*=−0.18) and anxiety (between group mean difference −0.9; 95% CI −1.6 to −0.2; *P*=.007; Cohen *d*=−0.21) |
| Buhrman et al [34] | No between-group difference in Multidimensional Pain Inventory over 12 weeks | —^i^ | Significant improvement in Coping Strategies Questionnaire subscale catastrophizing for EG over 12 weeks (*F*_1,48_=11.9; *P*=.0001) and Quality of Life (*F*_1,48_=10.8; *P*=.0002) |
| Calner et al [19] and Nordin et al [20] | No between-group difference in pain VAS^j^ at 4 months or 12 months | No between-group difference in function Pain Disability Index at 4 months or 12 months | Significant improvement in catastrophizing subscale of Coping Strategies Questionnaire for EG at 12 months (between-group mean difference −0.8; 95% CI −0.3 to −1.3; Cohen *d*=0.61; *P*=.001) |
| Carpenter et al [22] | No between-group difference in pain severity (pain rating—*average pain*, *lowest pain*, *highest pain*) at 3 weeks^a^ | Significant improvement in RMDQ^k^ for EG at 3 weeks (*F*_1,126_=6.65; *P*<.011; Cohen *d*=−0.45) | Significant improvement in Fear Avoidance Belief Questionnaire physical activity for EG at 3 weeks (*F*_1,126_= 25.14; *P*<.001, Cohen *d*=−0.80); Survey of Pain Attitudes scales−control (*F*_1,126_=44.65; *P*<.001, Cohen *d*=1.01), disability (*F*_1,126_=19.48; *P*<.001; Cohen *d*=−0.72), harm-exercise (*F*_1,126_=24.48; *P*<.001; Cohen *d*=−0.80), emotion (*F*_1,126_=−26.51; *P*<.001; Cohen *d*=0.82), medication (*F*=−28.24; *P*<.001; Cohen *d*=−0.88); self-efficacy (*F*_1,126_=33.97; *P*<.001; Cohen *d*=0.89), Pain Catastrophising Scale: rumination (*F*=11.30; *P*< 001; Cohen *d*=−0.59), magnification (*F*_1,126_=12.61; *P*<.001; Cohen *d*=−0.63), helplessness (*F*_1,126_=21.06; *P*<.001; Cohen *d*=−0.77), negative mood regulation (*F*_1,126_=17.31; *P*<.001; Cohen *d*=0.7) |
| Chhabra et al [36] | No between-group differences in pain NRS at 12 weeks | Significant improvement in Modified Oxford Disability Index for EG at 12 weeks (*F*_1,90_=9.053; *P*=.003; partial eta square=0.091) | Between-group analysis not undertaken for secondary outcome measures |
| Chiauzzi et al [23] | No between-group difference in Brief Pain Inventory at 3 and 6 months | No between-group difference in Oswestry Disability Index at 3 and 6 months | Significant improvement in stress subscale of Depression Anxiety Stress Scale for EG at 6 months (*t*=2.65; *P*<.05): coping subscale of Chronic Pain Coping Inventory (*t*=−2.44; *P*<.05): social support subscale (*t*=−2.89; *P*<.05), and Participants’ Global Impression of Change (*t*=2.83; *P*<.005) |
| Del Pozo-Cruz et al [16-18] | Significant improvement in pain/discomfort as measured in EuroQol – Five Dimensions – Three Levels Health Questionnaire for EG at 9 months (OR 7.652, 95% CI 2.480 to 23.613; *P*<.001) | Significant improvement in RMDQ for EG at 9 months (between group mean difference −8.42; 95% CI−9.76 to −7.07; *P*<.001; Cohen *d*=−2.8)  Significant improvement in functional disability in ODI for EG at 9 months (OR 5.420, 95% CI 1.707 to 17.216; *P*=.001) | Significant improvement in muscle endurance tests for lumbar extension for EG at 9 months (between group mean difference 18.50; 95% CI 9.13 to 27.86; *P*=.001; Cohen *d*=0.5): abdominals (between-group mean difference 18.64; 95% CI 9.62 to 27.65; *P*<.001; Cohen *d*=0.5); number of low back pain episodes (between-group mean difference −1.76; 95% CI −2.01 to −1.50; *P*<.001; Cohen *d*=−1.92): EuroQol – Five Dimensions – Three Levels Health Questionnaire (OR 3.587, 95% CI 2.210 to 5.823; *P*<.001), STarTBack Screening Tool score (OR 3.043, 95% CI 1.779 to 5.206; *P*< 0.001) |
| Irvine et al [24] | Significant improvement in current back pain questionnaire for EG1 (app) versus CG at 16 weeks (*P*=.02; eta square=0.043) and EG1 versus EG2 (alternative care; *P*=.016; eta square=0.016) | Significant improvement in functionality, quality of life, and well-being for EG1 versus CG at 8 weeks (*P*=.003; eta square=0.029) and 16 weeks (*P*=.001; eta square=0.033)  No between group difference between EG1 versus EG2 at 8 or 16 weeks | Significant improvement in patient activation for EG1 versus EG2, at 16 weeks (*P*=.027; eta square=0.02), worksite outcomes (*P*=.36; eta square=0.07), theory of planned care (*P*<.001; eta square=0.052), attitudes toward pain (*P*=.008; eta square=0.026), and prevention helping behaviors (*P*=.025; eta square=0.02) |
| Krein et al [25] | No between-group difference in level of pain severity at either 6 months or 12 months | Significant improvement in RMDQ for EG at 6 months (between group difference=1.6; 95% CI 0.3-2.8; *P*=.02)  No between-group differences in RMDQ at 12 months or Short form Medical Outcomes Study at either 6 or 12 months | — ^i^ |
| Marangoni [26] | Significant improvement in pain VAS post test (~ 16 days) for EG1 (CASP^l^) versus CG at 12 weeks (group mean difference 10.8; 95% CI 6.12 to 15.5; *P*=.000). Significant improvement for EG2 (FLIP^m^) versus CG (group mean difference 10.3; *P*=.000)  No between group differences for EG1 v EG2 | — ^i^ | — ^i^ |
| Mecklenberg et al [27] | Significant improvement in KOOS pain for EG at 12 weeks (between-group mean difference −7.7; 95% CI −12.3 to −3; *P*=.002)  Significant improvement in EG after 12 weeks for pain (VAS) (*P*=.001; between-group mean difference −12.3; 95% CI −19.1 to −5.4) | Significant improvement in KOOS– Physical Function for EG at 12 weeks (between-group mean difference −7.2; 95% CI −11.5 to −3; *P*=.001) | Significant improvement in stiffness Visual Analogue Scale for EG at 12 weeks (between-group mean difference −13.4; 95% CI −21.1 to −5.6; *P*=.001), surgery chance next year (between-group mean difference −9.4; 95% CI −16.6 to −2.2; *P*=.01), surgery chance in next 2 years (between-group mean difference −11.3; 95% CI −20.1 to −2.5; *P*=.01), surgery chance in next 5 years (between-group mean difference −14.6; 95% CI −23.6 to −5.5; *P*=.002), surgery interest (between-group mean difference −1.0; 95% CI −1.7 to −0.2; *P*=.01), and understanding of condition and treatment options (between-group mean difference 0.9; 95% CI 0.6 to 1.3; *P*>.001) |
| Peters et al [31] | No between-group difference in pain intensity for EG1 (internet-delivered cognitive behavioral therapy) versus CG, EG2 (psychology intervention) versus CG, or EG1 versus EG2 in pain intensity at post treatment or 6 months | No between-group difference in Fibromyalgia Impact Questionnaire for EG1 versus CG, EG2 versus CG, or EG1 versus EG2 at post treatment or 6 months | — ^i^ |
| Petrozzi et al [30] | No between-group difference in pain NRS post treatment, 6 and 12 months | No between-group difference for RMDQ post treatment, 6 and 12 months | — ^i^ |
| Shebib et al [28] | Significant difference in Modified Von Korff scale-pain for EG at 12 weeks (between-group mean difference −16.4; 95% CI −22 to −10.9; *P*=.001)  Significant improvement in VAS pain score for EG at 12 weeks (between-group mean difference −16; 95% CI −22.5 to −9.4; *P*=.001) | Significant improvement in Modified Von Korff scale-disability for EG at 12 weeks (between-group mean difference −13; 95% CI −19.3 to −6.7; *P*<.0001)  Significant improvement in Oswestry Disability Index for EG at 12 weeks (between-group mean difference −4.1; 95% CI −6.5 to −1.8; *P*<.001) | Significant improvement in Visual Analogue Scale impact on daily life score for EG at 12 weeks (between-group mean difference −11.8; 95% CI −19.3 to −4.3; *P*=.002); surgery interest (between-group mean difference −0.4; 95% CI −0.7 to −0.1; *P*=.01): understanding of condition and treatment options (between-group mean difference 0.5; 95% CI 0.2 to 0.7; *P*=.0005) |
| Toelle et al [35] | Significant improvement in pain NRS at 12 weeks (*P*=.021) | No between-group difference Hanover functional ability questionnaire | — ^i^ |
| Van den Heuvel et al [32] | No between-group differences for frequency or severity score of pain EG1 (computer program breaks and exercises) versus CG, EG2 (breaks and exercises) versus CG, and EG1 versus EG2 at 8 weeks | — ^i^ | — ^i^ |

^a^No between-group data for 6-week outcomes.

^b^See Multimedia Appendix 6 for the list of all abbreviations for outcome measures.

^c^EG: experimental group.

^d^CG: control group.

^e^WOMAC: Western Ontario and McMaster Universities Osteoarthritis Index

^f^NRS: Numeric rating scale

^g^KOOS: Knee Injury and Osteoarthritis Outcome Score

^h^HOOS: Hip Injury and Osteoarthritis Outcome Score

^i^Not measured

^j^VAS^:^ Visual Analogue Scale

^k^RMDQ: Roland Morris Disability Questionnaire

^l^CASP: computer-assisted stretching program.

^m^FLIP: facsimile lesson with instructional pictures.

References:

[6] Bennell KL, Nelligan R, Dobson F, Rini C, Keefe F, Kasza J, French S, Bryant C, Dalwood A, Abbott JH, Hinman RS. Effectiveness of an internet-delivered exercise and pain-coping skills training intervention for persons with chronic knee pain: a randomized trial. Ann Intern Med 2017 Apr 4; 166(7):453-62

[16] del Pozo-Cruz B, Parraca JA, del Pozo-Cruz J, Adsuar JC, Hill J, Gusi N. An occupational, internet-based intervention to prevent chronicity in subacute lower back pain: a randomised controlled trial. J Rehabil Med 2012 Jun; 44(7):581-7

[17] del Pozo-Cruz B, Gusi N, del Pozo-Cruz J, Adsuar JC, Hernandez-Mocholí M, Parraca JA. Clinical effects of a nine-month web-based intervention in subacute non-specific low back pain patients: a randomized controlled trial. Clin Rehabil 2013 Jan; 27(1):28-39

[18] del Pozo-Cruz B, Adsuar JC, Parraca J, del Pozo-Cruz J, Moreno A, Gusi N. A web-based intervention to improve and prevent low back pain among office workers: a randomized controlled trial. J Orthop Sports Phys Ther 2012 Oct; 42(10):831-41

[19] Calner T, Nordin C, Eriksson MK, Nyberg L, Gard G, Michaelson P. Effects of a self-guided, web-based activity programme for patients with persistent musculoskeletal pain in primary healthcare: a randomized controlled trial. Eur J Pain 2017 Jul; 21(6):1110-20

[20] Nordin CA, Michaelson P, Gard G, Eriksson MK. Effects of the web behavior change program for activity and multimodal pain rehabilitation: randomized controlled trial. J Med Internet Res 2016 Oct 5; 18(10):e265

[21] Allen KD, Arbeeva L, Callahan LF, Golightly YM, Goode AP, Heiderscheit BC, Huffman KM, Severson HH, Schwartz TA. Physical therapy vs internet-based exercise training for patients with knee osteoarthritis: results of a randomized controlled trial. Osteoarthritis Cartilage 2018 Mar; 26(3):383-96

[22] Carpenter KM, Stoner SA, Mundt JM, Stoelb B. An online self-help CBT intervention for chronic lower back pain. Clin J Pain 2012 Jan; 28(1):14-22

[23] Chiauzzi E, Pujol LA, Wood M, Bond K, Black R, Yiu E, Zacharoff K. painACTION-back pain: a self-management website for people with chronic back pain. Pain Med 2010 Jul; 11(7):1044-58

[24] Irvine AB, Russell H, Manocchia M, Mino DE, Glassen TC, Morgan R, Gau JM, Birney AJ, Ary DV. Mobile-web app to self-manage low back pain: randomized controlled trial. J Med Internet Res 2015 Jan 2; 17(1):e1

[25] Krein SL, Kadri R, Hughes M, Kerr EA, Piette JD, Holleman R, Kim HM, Richardson CR. Pedometer-based internet-mediated intervention for adults with chronic low back pain: randomized controlled trial. J Med Internet Res 2013 Aug 19; 15(8):e181

[26] Marangoni AH. Effects of intermittent stretching exercises at work on musculoskeletal pain associated with the use of a personal computer and the influence of media on outcomes. Work 2010; 36(1):27-37

[27] Mecklenburg G, Smittenaar P, Erhart-Hledik JC, Perez DA, Hunter S. Effects of a 12-week digital care program for chronic knee pain on pain, mobility, and surgery risk: randomized controlled trial. J Med Internet Res 2018 Apr 25; 20(4):e156

[28] Shebib R, Bailey JF, Smittenaar P, Perez DA, Mecklenburg G, Hunter S. Randomized controlled trial of a 12-week digital care program in improving low back pain. NPJ Digit Med 2019; 2:1

[29] Bennell KL, Nelligan RK, Rini C, Keefe FJ, Kasza J, French S, Forbes A, Dobson F, Abbott JH, Dalwood A, Harris A, Vicenzino B, Hodges PW, Hinman RS. Effects of internet-based pain coping skills training before home exercise for individuals with hip osteoarthritis (HOPE trial): a randomised controlled trial. Pain 2018 Sept; 159(9):1833-42

[30] Petrozzi MJ, Leaver A, Ferreira PH, Rubinstein SM, Jones MK, Mackey MG. Addition of MoodGYM to physical treatments for chronic low back pain: a randomized controlled trial. Chiropr Man Therap 2019; 27:54

[31] Peters ML, Smeets E, Feijge M, van Breukelen G, Andersson G, Buhrman M, Linton SJ. Happy despite pain: a randomized controlled trial of an 8-week internet-delivered positive psychology intervention for enhancing well-being in patients with chronic pain. Clin J Pain 2017 Nov; 33(11):962-75

[32] van den Heuvel SG, de Looze MP, Hildebrandt VH, Thé KH. Effects of software programs stimulating regular breaks and exercises on work-related neck and upper-limb disorders. Scand J Work Environ Health 2003 Apr; 29(2):106-16

[33] Bossen D, Veenhof C, van Beek KE, Spreeuwenberg PM, Dekker J, de Bakker DH. Effectiveness of a web-based physical activity intervention in patients with knee and/or hip osteoarthritis: randomized controlled trial. J Med Internet Res 2013 Nov 22; 15(11):e257

[34] Buhrman M, Nilsson-Ihrfeldt E, Jannert M, Ström L, Andersson G. Guided internet-based cognitive behavioural treatment for chronic back pain reduces pain catastrophizing: a randomized controlled trial. J Rehabil Med 2011 May; 43(6):500-5

[35] Toelle T, Utpadel-Fischler D, Haas K, Priebe J. App-based multidisciplinary back pain treatment versus combined physiotherapy plus online education: a randomized controlled trial. NPJ Digit Med 2019; 2:34

[36] Chhabra HS, Sharma S, Verma S. Smartphone app in self-management of chronic low back pain: a randomized controlled trial. Eur Spine J 2018 Nov; 27(11):2862-74
