# Supplementary material for: The Effectiveness of Digital Health Interventions in the Management of Musculoskeletal Conditions: Systematic Literature Review
Source: J Med Internet Res. 2020 Jun 5;22(6):e15617. doi: 10.2196/15617 (PMC7305565; doi:10.2196/15617)
Supplement: Multimedia Appendix 5 [file jmir_v22i6e15617_app5.docx]

**Multimedia Appendix 5**. **Cochrane Risk of Bias (ROB) assessment**

| Domain | Description | High ROB | Low ROB | Unclear ROB |
| --- | --- | --- | --- | --- |
| Random sequence generation | Described the method used to generate the allocation sequence in sufficient detail to allow an assessment of whether it should produce comparable groups | Selection bias (biased allocation to interventions) due to inadequate generation of a randomized sequence | Random sequence generation method should produce comparable groups | Not described in sufficient detail |
| Allocation concealment | Described the method used to conceal the allocation sequence in sufficient detail to determine whether intervention allocations could have been foreseen before or during enrolment | Selection bias (biased allocation to interventions) due to inadequate concealment of allocations prior to assignment | Intervention allocations likely could not have been foreseen in before or during enrolment | Not described in sufficient detail |
| Selective reporting | Stated how the possibility of selective outcome reporting was examined by the authors and what was found | Reporting bias due to selective outcome reporting | Selective outcome reporting bias not detected | Insufficient information to permit judgment |
| Other sources of bias | Any important concerns about bias not addressed above | Bias due to problems not covered elsewhere in the table | No other bias detected | There may be a risk of bias, but there is either insufficient information to assess whether an important risk of bias exists or insufficient rationale or evidence that an identified problem will introduce bias |
| Blinding (participants and personnel) | Described all measures used, if any, to blind study participants and personnel from knowledge of which intervention a participant received. Provided any information relating to whether the intended blinding was effective. | Performance bias due to knowledge of the allocated interventions by participants and personnel during the study. | Blinding was likely effective. | Not described in sufficient detail |
| Blinding (outcome assessment) | Described all measures used, if any, to blind outcome assessors from knowledge of which intervention a participant received. Provided any information relating to whether the intended blinding was effective. | Detection bias due to knowledge of the allocated interventions by outcome assessors. | Blinding was likely effective. | Not described in sufficient detail |
| Incomplete outcome data | Described the completeness of outcome data for each main outcome, including attrition and exclusions from the analysis. Stated whether attrition and exclusions were reported, the numbers in each intervention group (compared with total randomized participants), reasons for attrition/exclusions where reported. | Attrition bias due to amount, nature or handling of incomplete outcome data | Handling of incomplete outcome data was complete and unlikely to have produced bias | Insufficient reporting of attrition/ exclusions to permit judgment |

(Adapted from Cochrane Risk of Bias Tool [14])
