# Supplementary material for: The Effectiveness of Digital Health Interventions in the Management of Musculoskeletal Conditions: Systematic Literature Review
Source: J Med Internet Res. 2020 Jun 5;22(6):e15617. doi: 10.2196/15617 (PMC7305565; doi:10.2196/15617)
Supplement: Multimedia Appendix 6 [file jmir_v22i6e15617_app6.docx]

**Multimedia Appendix 6**. **List of abbreviations for outcomes measures**

| AIMS2 | Arthritis Impact Measurement Scale 2 | ASES | Arthritis Self-Efficacy Scale |
| --- | --- | --- | --- |
| AQoL-2 | Assessment of Quality of Life (version 2) | BPI | Brief Pain Inventory |
| COMM | Current Opioid Misuse Measure | CO-OP | Dartmouth Co-operative Functional Assessment Chart |
| CPCI | Chronic Pain Coping Inventory | CSS | Current Symptom Score |
| CSQ | Coping Strategies Questionnaire | DASS | Depression Anxiety Stress Scale |
| DCS | Decisional Conflict Scale | EQ-5D-3L | EuroQol – Five Dimensions – Three Levels Health Questionnaire |
| FABQ | Fear Avoidance Belief Questionnaire | FIQ | Fibromyalgia Impact Questionnaire |
| GCPS | Graded chronic pain scale | GSE | General Self-Efficacy Questionnaire |
| HADS | Hospital Anxiety and Depression Scale | HFAQ | Hanover functional ability questionnaire |
| heiQ | Health Education Impact Questionnaire | HOOS | Hip Injury and Osteoarthritis Outcome Score |
| KOOS | Knee Injury and Osteoarthritis Outcome Score | KOOS-PS | Knee Injury and Osteoarthritis Outcome Score Physical Function Short form |
| KPD-38 | Klinisch Psychologische Diagnosesystem 38 | Korff (pain) | Modified Von Korff scale (Pain) |
| Korff (disability) | Modified Von Korff scale (Disability) | MCS | Mental component summary score |
| MODI | Modified Oxford Disability Index | MOS | Short form Medical Outcomes Study 36 (SF-36) |
| MPI | Multidimensional Pain Inventory | MVES | Maximal Voluntary Extension Strength (neck) |
| MVFS | Maximal Voluntary Flexor Strength (neck) | NDI | Neck Disability Index |
| NMRS | Negative Mood Regulation Scale | NRS | Numeric Rating Scale |
| ODI | Oswestry Disability Questionnaire/Index | ODQ | Oswestry Disability Questionnaire |
| PA | Physical Activity | PAIRS | Pain and Impairment Relationship Scale |
| PAM | Patient Activation Measure | PASE | Physical Activity Scale for the Elderly |
| PCS | Pain Catastrophising Scale | PCSS | Pain Component Summary Score |
| PDI | Pain Disability Index | PDMS | Preparation for Decision Making Scale |
| PGIC | Participants’ Global Impression of Change | PHQ-8 | Personal Health Questionnaire Depression Scale |
| PSA | Pain Spot Assessment | PSEQ | Pain Self-Efficacy Questionnaire |
| QOLI | Quality of Life Inventory | RMDQ | Roland Morris Disability Questionnaire |
| SBST | STarTBack Screening Tool | SCS-SF | Self-Compassion Scale |
| SF-36 | Short form Medical Outcomes Study 36 (SF-36) | SOPA | Survey of Pain Attitudes |
| SP | Stanford Presenteeism Scale | SQAHEPA | Short Questionnaire to Assess Health-Enhancing Physical Activity |
| TPB | Theory of Planned Behaviour Constructs | TUG | Timed Up and Go |
| UCLA | University of California Los Angeles activity scale scores | VAS | Visual Analogue Scale |
| VR-12 | Veterans RAND 12-item health survey | WAI | Work Ability Index |
| Web-BCPA | Web Behaviour Change Program for Activity | WLQ | Work Limitation Questionnaire |
| WOMAC | Western Ontario and McMaster Universities Osteoarthritis Index | WRFQ | Work Role Functioning Questionnaire |
